# Changes in Health Care Use Among Undocumented Patients, 2014-2018

**DOI:** 10.1001/jamanetworkopen.2021.0763

**Published:** 2021-03-05

**Authors:** Joseph Nwadiuko, Jashalynn German, Kavita Chapla, Frances Wang, Maya Venkataramani, Dhananjay Vaidya, Sarah Polk

**Affiliations:** 1 Department of Medicine, University of Pennsylvania Perelman School of Medicine, Philadelphia; 2 Leonard Davis Institute of Health Economics, University of Pennsylvania, Philadelphia; 3 Fielding School of Public Health, Department of Health Policy and Management, University of California, Los Angeles; 4 Department of Medicine, Johns Hopkins School of Medicine, Baltimore, Maryland; 5 Division of Endocrinology, Department of Medicine, Duke University School of Medicine, Durham, North Carolina; 6 Department of Pediatrics, Johns Hopkins School of Medicine, Baltimore, Maryland; 7 Johns Hopkins Center for Health/Salud and Opportunity for Latinos, Baltimore, Maryland

## Abstract

**Importance:**

The 2016 presidential campaign was marked by intensified rhetoric around the deportation of undocumented immigrants. The association of such rhetoric with primary, emergency, and inpatient care among undocumented immigrants is unclear.

**Objective:**

To examine the association of increased anti-immigrant rhetoric during the 2016 presidential campaign with health care use among a group of Medicaid-ineligible patients largely composed of undocumented immigrants.

**Design, Setting, and Participants:**

Using a difference-in-differences (DID) approach, this cohort study analyzed health care use between January 1, 2014, and May 31, 2018, in a retrospective cohort of Medicaid and Medicaid-ineligible (>90% undocumented) adult and pediatric patients. The inflection point of interest was June 16, 2015, the date of Donald Trump’s announcement of candidacy, which represented a documented increase in anti-immigration rhetoric during the presidential campaign. Analyses were controlled for age, self-reported sex, and baseline comorbidities. Data analysis was conducted from August 28, 2018, to September 1, 2020.

**Main Outcomes and Measures:**

The DID of the number of completed primary care encounters before and after June 16, 2015, in Medicaid compared with Medicaid-ineligible patients. Secondary outcomes included the DID of emergency department (ED) visits and inpatient discharges over the same period.

**Results:**

There were 20 211 patients included in the analysis: 1501 (7.4%) in the sample of predominantly undocumented Medicaid-ineligible patients (861 [57.4%] female) and 18 710 (92.6%) in the Medicaid control group (10 443 [55.8%] female). The mean (SD) age as of 2018 in the Medicaid-ineligible group was 38.2 (15.4) years compared with 22.2 (16.5) years in the control group. There was a differential decrease in completed visits among Medicaid-ineligible children compared with Medicaid children (DID estimate, 0.8; 95% CI, 0.7-0.9) and Medicaid-ineligible adults (DID estimate, 0.8; 95% CI, 0.8-0.9). There was also a significant differential increase in ED visits among Medicaid-ineligible children (DID estimate, 2.3; 95% CI, 1.1-5.0). In addition, there was a differential decrease in inpatient discharges among Medicaid-ineligible adults (DID estimate, 0.5; 95% CI, 0.4-0.7), with no significant change in ED visits or ED admission rates in this group.

**Conclusions and Relevance:**

In this cohort study, there was a significant decrease in primary care use among undocumented patients during a period of increased anti-immigrant rhetoric associated with the 2016 presidential campaign, coincident with an increase in ED visits among children and a decrease in inpatient discharges among adults, with the latter possibly attributed to a decrease in elective admissions during this period.

## Key Points

**Question:**

Did health care use among undocumented residents change with increasing anti-immigrant rhetoric during the 2016 presidential campaign?

**Findings:**

In this cohort study based on a single-center analysis of health care use among 20 211 adults and children, there was a 34.5% decrease in completed primary care visits among undocumented children and 43.3% decrease in completed primary care visits among undocumented adults between June 16, 2015 (the start of the Trump campaign for presidency, associated with an increase in anti-immigrant rhetoric), and May 31, 2018, which was significantly greater than patients in the Medicaid group.

**Meaning:**

A significant decrease was noted in this cohort study in the use of primary health care services among a sample of predominantly undocumented patients compared with those in a Medicaid control group in the setting of rising anti-immigrant rhetoric associated with the 2016 presidential campaign.

## Teaser

This cohort study examines the changes in use of health care services among undocumented immigrants during increased anti-immigrant rhetoric before the 2016 presidential election.

## Introduction

Approximately 11 million undocumented immigrants live in the US.^1^ Undocumented populations face substantial, multifactorial barriers to health care, including lack of insurance, racism, limited English proficiency, complex and unfamiliar health systems, transportation, and lower household incomes.^2^ Even when available, health care use may be suppressed by both policy-driven changes in immigration enforcement levels^3,4^ and fluctuations in anti-immigrant sentiment.

The association between restrictive or unfavorable immigration policies and health care and social service use, also known as the chilling effect, has been well documented. In a study of US national Medicaid registration data from 1992 to 2003, decreases in Medicaid participation among citizen children of noncitizen parents corresponded to spikes in immigration enforcement.^5^ Decreases and delays in initiation of prenatal care in North Carolina and Arizona followed both states’ adoption of provisions increasing local law enforcement cooperation with federal immigration officials.^6,7^ More recently, the expansion of the definition of public charge by US Citizenship and Immigration Services as a condition of inadmissibility for permanent residence visas is expected to deter eligible families from accessing public benefits.^8^

Generalized anti-immigrant sentiment may raise another potential barrier to health care use, particularly because it was a pronounced issue during the 2015-2016 US presidential campaign period. Examples of immigrant-directed harassment during the 2015-2016 pre-election period have been documented.^9^ In Maryland, hate crimes increased by 31.0% between 2014 and 2015 and by another 45.8% again between 2015 and 2016, with most associated with the victim’s race or ethnicity.^10^ Chu et al^11^ analyzed the association between anti-immigration rhetoric during the pre-2016 election period and prenatal care, finding in their study sample of approximately 17 000 women who had given birth between August 2011 and July 2017 a significant increase in days until the first prenatal visit and a decrease in both total prenatal visits and mean hemoglobin level (an indicator of inadequate prenatal care) among Latina immigrants, coincident to a pre-election inflection of Google search terms such as Make America Great Again, Mexico wall, and deportation. To our knowledge, there has been no similar analysis examining health care use generally among undocumented immigrant populations during the 2015-2016 election campaign.

We analyzed health care use in a retrospective cohort comprising both Medicaid-ineligible (predominantly undocumented) patients participating in a mid-Atlantic health system’s charity and sliding scale program and Medicaid-insured patients of the same health system between January 1, 2014, and May 31, 2018. Using a difference-in-differences (DID) approach, we investigated changes in use of ambulatory, emergency, and inpatient health care services in both groups with particular interest in changes in use among undocumented patients after June 16, 2015, hypothesizing a decrease in ambulatory care and increase in emergency and inpatient care afterwards. This date corresponds to the date of President Trump’s presidential campaign announcement and an inflection point in terms of anti-immigrant rhetoric associated with the presidential campaign, with possible associations with undocumented immigrant health care use, as noted previously by Chu et al.^11^

## Methods

The study was approved by the Johns Hopkins School of Medicine Institutional Review Board, Data Trust Council, and Johns Hopkins Community Physicians Research Subcommittee; we also obtained a Certificate of Confidentiality from the National Institute of Minority Health and Health Disparities. A waiver of consent was granted by the institutional review board because the study was retrospective and only involved record review. This study followed the Strengthening the Reporting of Observational Studies in Epidemiology (STROBE) reporting guideline for cohort studies.

### Analytical Assumptions

A number of factors may be associated with a patient’s ability to present themselves for care, including, but not limited to, educational level, income, insurance access, transportation, cultural sensitivity, and the patient’s perceptions of their health.^12^ We assumed based on prior evidence that undocumented residents in particular have similar factors in care-seeking but face unique barriers to care based on their undocumented status and particularly to the deterrence of immigration enforcement.^2^ As President Trump’s candidacy represented a sudden, exogenous increase in the perceived salience of immigration enforcement, we assumed that changes in the use of health care services among the Medicaid-ineligible group would otherwise not have changed except for his candidacy and eventual election.

### Sample Selection

All patients were drawn from outpatient clinics in the Johns Hopkins Health System (JHHS). Medicaid-ineligible patients were identified by their participation in 1 of 2 programs. The first program is The Access Partnership (TAP), a fee-waiver program started by the JHHS in 2009 to provide care for low-income, Medicaid-ineligible patients. Individuals are eligible for the program if they reside in 1 of 10 zip codes in Baltimore City and have a household income of less than 200% of the federal poverty line. TAP participants receive coverage for primary care, procedures, hospitalizations, and specialty care at the Johns Hopkins Hospital and Johns Hopkins Bayview Medical Center for a quarterly fee of $20. Since the passage of the Patient Protection and Affordable Care Act and expansion of Medicaid within Maryland (effective January 1, 2014), undocumented immigrants have come to represent 92% of TAP participants according to internal documentation. The second program is a sliding scale access program for uninsurable, low-income patients. These patients are seen at a Johns Hopkins–affiliated ambulatory practice. Since Medicaid expansion in 2014, more than 90% of these patients have been undocumented. During the time covered by this study, only Washington, Oregon, California, New York, Massachusetts, Illinois, and the District of Columbia offered Medicaid to undocumented immigrants, with all those states except the District of Columbia offering access exclusively to children.^13^

Patients were included in the Medicaid-ineligible group if they had at least 2 scheduled encounters covered by TAP or the sliding scale at 1 of the 8 JHHS-affiliated medical, pediatric, or medicine-pediatric practices that accepted TAP and/or sliding scale between January 1, 2014, and October 31, 2015, and had no encounters paid for by any other insurer during the study period. Selection was done without knowledge of individual immigration status to protect the confidentiality of study patients. Patients were included in the control group if they had at least 2 scheduled encounters in these clinics covered by 1 of the 8 Medicaid-related managed care organizations licensed in the State of Maryland or Emergency Medicaid between January 1, 2014, and October 31, 2015, and no encounters paid for by any insurers other than Medicare, TAP, or the sliding scale plan during the course of the study, assuming that there was a small proportion of patients who briefly remained on TAP or sliding scale plans after Medicaid was expanded to cover adults on January 1, 2014. Patients were excluded from the study if they died before June 16, 2015, or had any visits paid by private insurance; patients of all ages were included. All patient data were derived from JHHS electronic medical record extracts of individual encounters and covered the period between January 1, 2014, and May 31, 2018. A flowchart detailing the selection process is presented in eFigure 1 in the Supplement.

### Outcomes

The primary outcome was the DID in the number of completed primary care encounters per 100 individuals per year between January 1, 2014, until June 16, 2015, and June 17, 2015, until May 31, 2018, between Medicaid-ineligible and Medicaid patients. Secondary exploratory outcomes were the DID in nonobstetric emergency department (ED) visits and inpatient discharges per 100 individuals during the same period.

The exposure variable of interest was undocumented status, which was represented by membership in the JHHS TAP/sliding scale program, with Medicaid beneficiaries as a control group. Children and adults were analyzed separately owing to unbalanced age distribution between the Medicaid and Medicaid-ineligible groups (Table 1), as well as distinct predicted changes in the use of outpatient services. For pediatric changes, we included children between the ages of 6 and 18 years as of 2018 (the end of our observation period) to exclude anticipated age-based variations in use related to changes in well-child visit frequency recommendation (after age 2 years) and drop-off in annual visits among adolescents (after age 14 years).^14^ Adults were defined as those older than 18 years as of 2018. For all outcomes, we produced adjusted changes of the mean number of encounters per 100 patients per quarter.

**Table 1.** Study Population

Variable	Population, No. (%)					
	Medicaid ineligible^a^			Medicaid		
	Total (N = 1501)	Adults (n = 1336)^b^	Children (n = 165)^c^	Total (N = 18 710)	Adults (n = 8904)^b^	Children (n = 10 616)^c^
2018 Age, mean (SD), y	38.2 (15.4)	41.3 (13.4)	13.7 (3.6)	22.2 (16.5)	36.4 (16.0)	11.3 (3.7)
Sex						
Female	861 (57.4)	782 (58.5)	79 (47.9)	10 443 (55.8)	5247 (64.8)	5196 (48.9)
Male	640 (42.6)	554 (41.5)	86 (52.1)	8267 (44.2)	2847 (35.2)	5420 (51.1)
Race						
American Indian, Alaska Native, Native Hawaiian, or Pacific Islander	2 (0.1)	2 (0.1)	0	38 (0.2)	16 (0.2)	22 (0.2)
Asian	11 (0.7)	11 (0.8)	0	52 (0.3)	21 (0.3)	31 (0.3)
Black	156 (10.4)	155 (11.6)	1 (0.6)	15 114 (80.8)	7070 (87.4)	8044 (75.8)
White	72 (4.8)	71 (5.3)	1 (0.6)	1040 (5.6)	622 (7.7)	418 (3.9)
Other or unknown	1260 (83.9)	1097 (82.1)	163 (98.8)	2466 (13.2)	365 (4.5)	2101 (19.8)
Ethnicity						
Hispanic/Latino	1188 (79.2)	1036 (77.6)	152 (92.1)	2281 (12.2)	301 (3.7)	1980 (18.7)
Diagnoses						
Asthma	77 (5.1)	56 (4.2)	21 (12.7)	5883 (31.4)	2400 (29.7)	3483 (32.8)
COPD	2 (0.1)	2 (0.2)	0	766 (4.1)	766 (9.5)	0
Diabetes	258 (17.2)	256 (19.2)	2 (1.2)	1905 (10.2)	1830 (22.6)	75 (0.7)
Hypertension	256 (17.1)	252 (18.9)	4 (2.4)	3194 (17.1)	3043 (37.6)	151 (1.4)
Depression	198 (13.2)	192 (14.4)	6 (3.6)	2491 (13.3)	2225 (27.5)	266 (2.5)

Abbreviation: COPD, chronic obstructive pulmonary disease.

^a^ As defined by income below 200% of the federal poverty line and unable to meet Maryland Medicaid eligibility guidelines and further meeting criteria for either the Johns Hopkins Health System charity or sliding scale programs.

^b^ Age greater than 18 years as of 2018.

^c^ As defined by ages between 6 and 18 years as of 2018.

We included several supplementary analyses and robustness checks. First, to better understand the factors associated with shifts in completed visit changes, we analyzed the DID and adjusted trends for no-show or cancellation rates and all scheduled appointments (ie, completed and noncompleted). Second, care seeking for some patients might be episodic and time limited, leading to artificial decreases timed around the end of our eligibility period (October 31, 2015) for patients who initiated care not long before that date. Therefore, we repeated the DID estimates for clinic visits with the sample isolated to those who had a JHHS scheduled appointment in 2014. Third, to better explain the association between ED visits and inpatient discharge trends, we calculated the DID of ED admission rates. Fourth, all primary and secondary outcomes were reestimated among all children up to age 18 years (as of 2018). Fifth, because changes in the primary outcome might be associated with changes in acute morbidity, we repeated the DID estimates for clinic visits for those who did not have any hospitalizations during the primary period. Sixth, because Hispanic/Latino immigration to Baltimore is relatively recent and composed largely of adult undocumented immigrants,^15,16^ we analyzed the DID estimates for clinic visits for Hispanic/Latino children who receive Medicaid compared with non–Hispanic/Latino children who receive Medicaid (with the assumption that children in the first group were born in the US and are in mixed-status families). Seventh, to minimize the patterns of Medicare recipients on our estimate, we analyzed the DID estimates for clinic visits for adults between the ages of 18 and 69 years as of 2018 (ie, those who did not reach Medicare age eligibility of 65 years during the study period). Eighth, we analyzed the DID estimates for clinic visits with a gaussian instead of a negative binomial distribution to rule out artificial changes in estimates generated by the choice of statistical model.

### Statistical Analysis

Data analysis was conducted from August 28, 2018, to September 1, 2020. Owing to observed overdispersion in the outcome variables (ie, variance greater than the mean), we calculated DID and quarterly trend estimates using an estimation equation with a negative binomial distribution with a log-link function. The exception to this method was our analysis of no-show and cancellation rates, which were not overdispersed and were estimated with a Poisson distribution. Robust SEs were clustered at the individual level, and we assumed an exchangeable correlation structure. All models were adjusted for year of birth, self-reported sex, and Elixhauser comorbidity index scores drawn from the first year of data from each individual. These covariates were chosen given the evidence of age- and sex-dependent differences in health care use^14,17^ as well as known immigrant health advantages compared with the US-born population that might also affect the use of health care services.^18^ The Elixhauser comorbidity index is a widely used comorbidity classification system developed for use with administrative data and has been validated as a predictor of inpatient mortality and health care use outcomes.^19,20,21^ Our model is represented by the following formula:

ln(*μ_ij_*) = *β_0_* + *β_1_*Cohort*_i_* + *β_2_*Prepost*_ij_* + *β_3_*Cohort*_i_* × Prepost*_ij_* + *β_4_*BirthYear*_i_* + *β_5_*Gender*_i_* + *β_6_*Elixhauser*_i_* + *β_7_*Gender*_i_* × Prepost*_ij_* + *β_8_*BirthYear*_i_* × Prepost*_ij_* + ln(time*_ij_*),

where the outcome μ specifies the number of encounters, the *i* subscript refers to each individual, and the *j* subscript specifies the period (ie, pre- or postannouncement). The offset for no-show and cancellation rates is *ln(number of scheduled visits)* instead of *ln*(*time).*The coefficient of interest was β_3_, the DID estimate. The method of generating quarterly trend estimates is similar and detailed in the eAppendix in the Supplement. All *P* values were determined using 2-sided tests, and results were deemed statistically significant at *P* < .05. All data were analyzed with Stata, version 15 (StataCorp LLC).

## Results

There were 20 211 patients in the sample (1501 [7.4%] in the predominantly undocumented Medicaid-ineligible group and 18 710 [92.6%] in the Medicaid control group). The Medicaid-ineligible group comprised 861 females (57.4%) and 640 males (42.6%) compared with 10 443 females (55.8%) and 8267 males (44.2%) in the control group. The mean (SD) age as of 2018 was 38.2 (15.4) years in the Medicaid-ineligible group compared with 22.2 (16.5) years in the control group. A further age breakdown is provided in eTable 1 in the Supplement. A total of 1188 individuals (79.2%) in the cohort group were Hispanic/Latino individuals compared with 2281 (21.2%) in the control group.

### Primary Care Use

**Figure 1.**
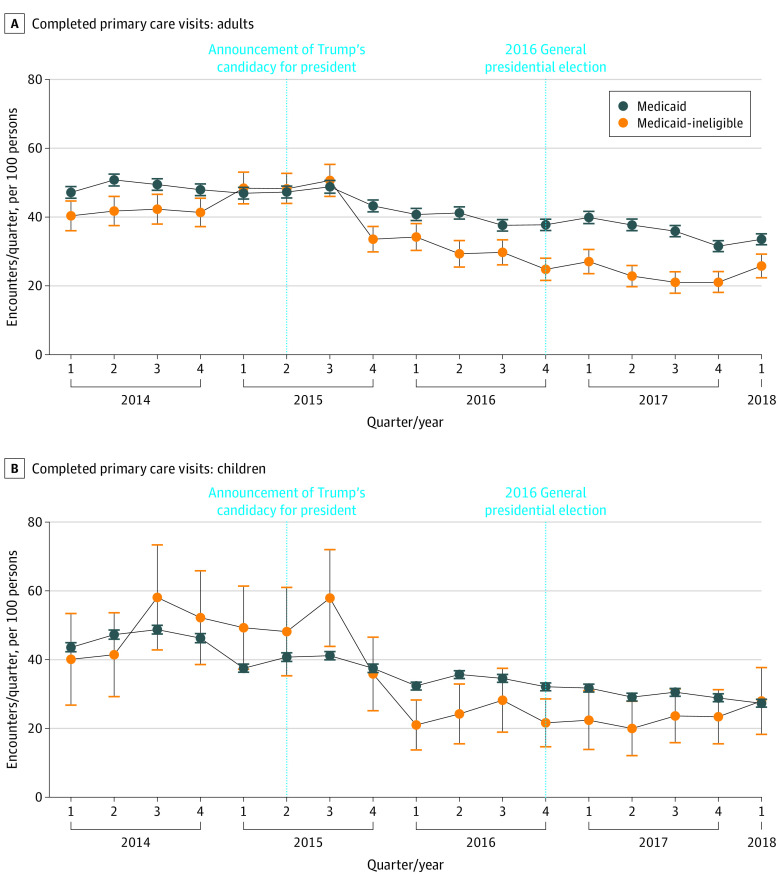
Adjusted Mean Number of Completed Primary Care Quarterly Visits Completed primary care visits for adults (A) and children (B). Error bars show 95% CIs.

We analyzed a total of 336 466 primary care appointments across 8 clinics. Unadjusted estimates of all baseline and outcome measures are provided in eTable 10 in the Supplement. At baseline, there was an adjusted annual mean for adults of 178.6 (95% CI, 169.9-187.4) completed primary care visits per 100 individuals in the Medicaid-ineligible group and 193.6 (95% CI, 189.7-197.5) visits per 100 individuals in the Medicaid group. For children, there was an adjusted baseline mean of 192.1 (95% CI, 171.8-212.4) completed visits per 100 individuals in the Medicaid ineligible group and 172.9 (95% CI, 170.5-175.3) completed visits per 100 individuals in the Medicaid group. After June 16, 2015, there was a differential decrease among Medicaid-ineligible children (adjusted postperiod mean, 108.9; −43.3%) compared with Medicaid children (adjusted postperiod mean, 126.3; −27.0%) (DID estimate, 0.8; 95% CI, 0.7-0.9) and Medicaid-ineligible adults (adjust postperiod mean, 116.9; −34.5%) compared with Medicaid adults (adjust postperiod mean, 1553; −19.7%) (DID estimate, 0.8; 95% CI, 0.8-0.9) (Table 2 and Figure 1). These estimates remained significant when restricted to patients with visits in 2014 (DID estimate for adults, 0.7; 95% CI, 0.7-0.8; DID estimate for children, 0.6; 95% CI, 0.5-0.8) (eTable 2 in the Supplement), when including all patients between ages 0 and 18 years as of 2018 (DID estimate, 0.8; 95% CI, 0.7-0.9) (eTable 9 in the Supplement), when restricted to patients without hospitalizations (DID estimate for adults, 0.8; 95% CI, 0.7-0.9; DID estimate for children, 0.8; 95% CI, 0.7-1.0) (eTable 3 in the Supplement), when restricted to patients between ages 19 and 69 years as of 2018 (DID estimate, 0.8; 95% CI, 0.8-0.9) (eTable 4 in the Supplement), and when analyzed with a gaussian distribution (DID estimate for adults, −0.2; 95% CI, −0.2 to −0.1; DID estimate for children, −0.3; 95% CI, −0.5 to −0.1) (eTable 5 in the Supplement). There was no significant difference in clinic use changes between Hispanic/Latino children recipients of Medicaid and non–Hispanic/Latino Medicaid children recipients (DID estimate, 1.0; 95% CI, 1.0-1.0) (eTable 6 in the Supplement). There was a statistically significant differential decrease in scheduled visits for Medicaid-ineligible adults and children (eTable 7, eFigure 2, and eFigure 3 in the Supplement). No-show and cancellation rates for Medicaid-ineligible patients increased nonsignificantly for adults and children, peaking after the 2016 election (eTable 7, eFigure 4, and eFigure 5 in the Supplement).

**Table 2.** Difference in Differences of Health Care Use After June 16, 2015

Variable	Annual encounters per 100 people, mean (95% CI), No.				Difference in differences	
	Medicaid-ineligible patients		Medicaid patients			
	Pre	Post	Pre	Post	IRR (95% CI)	*P* value
**Completed primary care visits**						
Adults	178.6 (169.9-187.4)	116.9 (108.7-125.1)	193.6 (189.7-197.5)	155.3 (151.2-159.4)	0.8 (0.8-0.9)	<.001
Children	192.1 (171.8-212.4)	108.9 (92.5-125.2)	172.9 (170.5-175.3)	126.3 (124.3-128.3)	0.8 (0.7-0.9)	.003
**Emergency department visits**						
Adults	14.8 (11.9-17.8)	14.8 (12.3-17.4)	51.2 (47.7-54.7)	49.0 (46.0-51.9)	1.1 (0.9-1.3)	.66
Children	5.8 (1.7-9.9)	13.1 (6.8-19.3)	27.3 (26.1-28.5)	26.6 (25.5-27.7)	2.3 (1.1-5.0)	.03
**Inpatient discharges**						
Adults	6.7 (5.3-8.2)	2.9 (2.2-3.7)	8.1 (7.4-8.8)	7.3 (6.67-7.95)	0.5 (0.4-0.7)	<.001
Children	2.6 (0-5.5)	2.8 (0-6.1)	2.0 (1.8-2.3)	1.8 (1.6-2.0)	1.2 (0.3-5.9)	.67

Abbreviation: IRR, incident rate ratio.

### ED Visits and Hospitalization 

We analyzed a total of 43 778 ED discharges and 10 975 inpatient discharges across 2 hospitals during the study period. At baseline, there was an adjusted annual mean of 14.8 (95% CI, 11.9-17.8) ED visits per 100 adults, 5.8 (95% CI, 1.7-9.9) ED visits per 100 children, 6.7 (95% CI, 5.3-8.2) inpatient discharges per 100 adults, and 2.6 (95% CI, 0-5.5) inpatient discharges per 100 children among Medicaid-ineligible patients (Table 2). Over the same period among Medicaid beneficiaries, there was an adjusted annual mean of 51.2 (95% CI, 47.7-54.7) ED visits per 100 adults, 27.3 (95% CI, 26.1-28.5) ED visits per 100 children, 8.1 (95% CI, 7.4-8.8) inpatient discharges per 100 adults, and 2.0 (95% CI, 1.8-2.3) inpatient discharges per 100 children.

**Figure 2.**
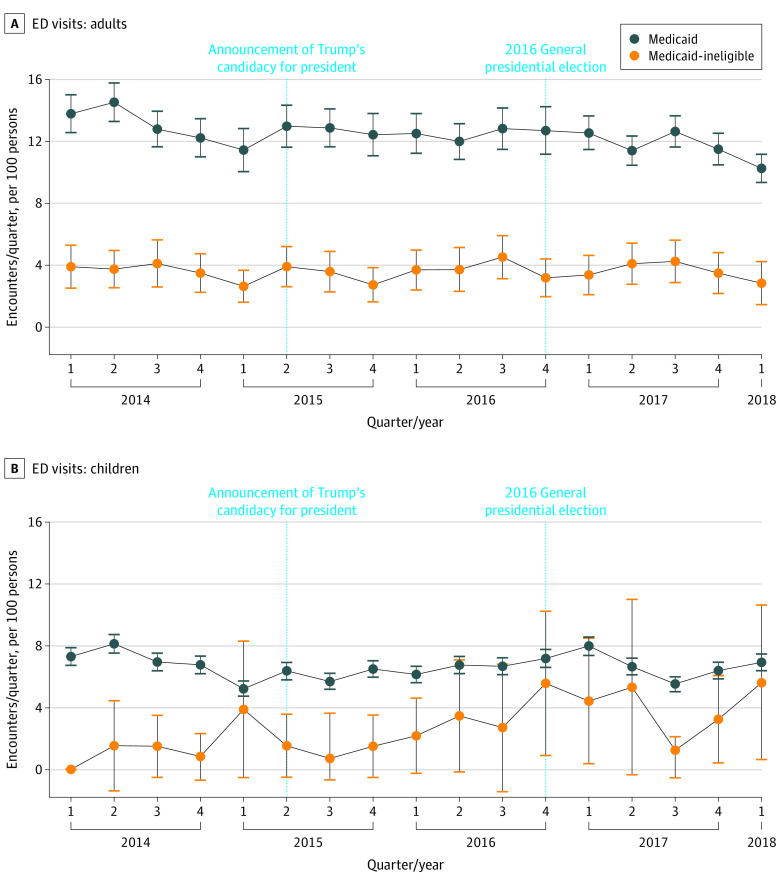
Adjusted Mean Number of Quarterly Emergency Department (ED) Visits Emergency department visits for adults (A) and children (B). Error bars show 95% CIs.

**Figure 3.**
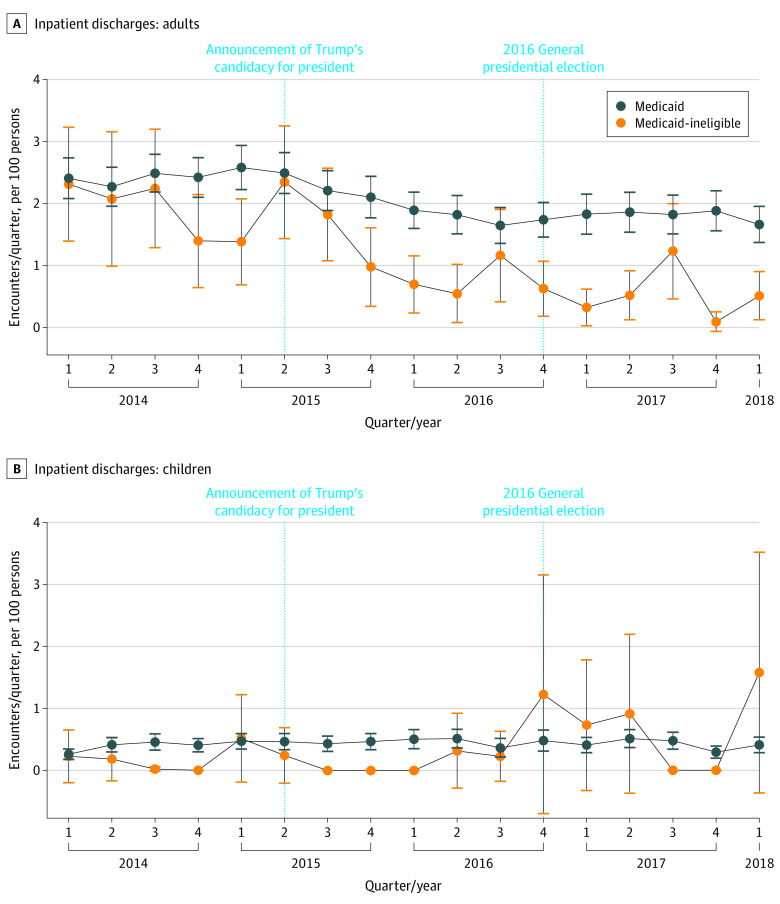
Adjusted Mean Number of Quarterly Inpatient Discharges Inpatient discharges for adults (A) and children (B). Error bars show 95% CIs.

Compared with data from before June 16, 2015, there was a significant differential increase in ED visits among Medicaid-ineligible children compared with Medicaid children (DID estimate, 2.3; 95% CI, 1.1-5.0) (Table 2 and Figure 2). There was no significant DID in ED visit changes among adults. There was a significant differential decrease in inpatient discharges among Medicaid-ineligible adults (DID estimate, 0.5; 95% CI, 0.4-0.7) but not children (Table 2 and Figure 3). There was no significant DID in ED admission rate changes during the study period (eTable 8 in the Supplement).

## Discussion

We found a significant decrease in the number of completed primary care visits among a group of predominantly (>90%) undocumented patients after the second quarter of 2015 compared with Medicaid patients. This period corresponded to a period of intensified anti-immigration rhetoric. Overall, these results are consistent and concurrent with the decrease in the use of prenatal care witnessed in Houston, Texas, among 12 569 immigrant Latina women starting in July 2015 and adds evidence to the hypothesis that anti-immigrant rhetoric during the 2016 election may have influenced health care use among undocumented patients.^11^ Decreases in the use of health care services have been reported beyond Baltimore and Houston, such as in Illinois, where the number of undocumented Medicaid beneficiaries decreased by 8.3% between June 30, 2015, and June 30, 2017, compared with an unchanged decrease of 2.6% among other beneficiaries.^22^

In the case of our analysis, the decrease in health care use appears to be associated with the chilling effect of anti-immigrant rhetoric. The change itself may be attributed to rhetoric-induced hypervigilance (ie, increased anticipation of being a target of discrimination), which has been well documented.^23^ A large number of undocumented immigrants in the region served by the health system fled from violence in Guatemala, Honduras, and El Salvador. As a result of destabilization in those nations, many of these immigrants become fearful of legal authorities and have symptoms of posttraumatic stress disorder, including hypervigilance.^24^ This hypervigilance is accentuated by racist harassment in the US community as well as routine questioning regarding nationality and citizenship status during registration at some health facilities. As such, even in the absence of a clear upsurge of community immigration enforcement, the sudden and substantial shift in anti-immigrant and anti-immigration campaign rhetoric could lead them to avoid all but the most necessary of health care encounters. Residing in a so-called immigrant-friendly city may do little to curb fear; even in California (considered a sanctuary state), only 4% of undocumented immigrants considered hospitals a safe space and delayed ED visits by a median of 2 to 3 days owing to concern of being apprehended by immigration services.^25,26^ The lack of changes in the use of health care services in Hispanic/Latino (documented immigrant and US citizen) Medicaid recipients might indicate the specificity of these effects on undocumented immigrants, although the fear of deportation of family members has been noted in other work to be associated with psychological harms in this population.^4,27,28^

The decrease in scheduled primary care appointments among Medicaid-ineligible patients largely stabilized in the fall of 2016, representing a basement effect in health care use in the Medicaid-ineligible population, although the increase in no-show rates for those visits after the 2016 election might point to continued chilling effects due to feared immigration changes. The steady decrease in use of primary care services among Medicaid patients is consistent with estimates showing decreases in primary care use in this group.^29^

The decrease in primary care use among Medicaid-ineligible patients may not be explained by concurrent unrelated phenomena, such as a switch in preference in hospital systems or migration from the Baltimore region. There were no new benefit programs for undocumented patients in the region and no change in access or cost to patients within TAP in this period; this time was also marked by an increase in the number of Hispanic/Latino individuals in Baltimore City by 4000.^30^ There were no increases in immigration-related arrests in the Baltimore region during the period of this decrease per Immigration and Customs Enforcement records released through the Freedom of Information Act to the Transactional Records Access Clearinghouse^31^ (eFigure 6 in the Supplement). When restricted to patients with visits in 2014, the estimates remained significant, signifying they are not completely explained by attrition of TAP members who initiated care in 2015 and discontinued it near the end of the study’s eligibility period (October 15, 2015). In addition, decreases in primary care visits among children cannot be fully accounted for by age-related changes in the use of pediatric care because we restricted our population to ages in which we would anticipate the lowest age-related decrease and controlled for the interaction of age with time in our statistical model.

The decrease in the use of primary care services likely has meaningful clinical consequences given that fear of deportation is associated with higher rates of cardiometabolic risk factors, such as increased blood pressure, that need and are amenable to early primary care intervention.^32^ Health care access is also necessary to maintain the high vaccination rates among children needed to sustain herd immunity against infectious diseases, such as measles. Primary care access for Latino populations is essential during the present coronavirus disease 2019 pandemic; among all racial and ethnic groups at JHHS, the Latino population has the highest severe acute respiratory syndrome coronavirus 2 test positivity rate.^33^

We believe the increase in ED visits among Medicaid-ineligible children is associated with either the morbidity related to delayed care seeking or substitution for office-based primary care; further investigation is needed. Either scenario has implications for the needs of undocumented minors in EDs. The decrease in inpatient visits in Medicaid-ineligible adults is not related to a significant differential decrease in ED use or ED admission rates. We believe it might be associated with a decrease in elective admissions, which might be associated with precedent outpatient visits. Further investigation is needed.

### Strengths and Limitations

There are several limitations to this study. First, it was an observational study limited to a single health system in a welcoming city with a relatively low number of undocumented immigrants; although we attempted to minimize confounding, it could not be eliminated. Second, we did not request undocumented status directly to preserve the privacy of study participants, which could hinder the precision of our estimates. Third, the data set does not use strictly comparable samples: aside from slightly healthier profiles among the Medicaid-ineligible sample, eligibility for TAP included those with incomes up to 200% of the federal poverty line (as opposed to Medicaid, which accepts only those with incomes less than 138% of the federal poverty line). Fourth, we cannot completely rule out attrition due to loss of eligibility for both coverage programs, although only 1.3% of completed clinic visits in our sample did not have associated coverage.

However, the strength of this study is its identification of a predominantly undocumented population of patients, in contrast from many studies that have focused on health care use outcomes among the Hispanic population as a whole, as well as a detailed analysis of health care encounters rather than patient-reported adherence. Ours is a hypothesis-generating study; as such, outcomes need to be confirmed in other settings with quantitative and qualitative work. There is also need for further work to be done regarding the worsening rhetoric and policy on health care utilization, including the announcements of Immigration and Customs Enforcement raids (including in Baltimore) during July 2019,^34^ October 2019 changes to the US Citizenship and Immigration Service’s public charge determination, and bans on immigration and visa approvals during the beginning of the coronavirus disease 2019 pandemic. In addition, more effort should be invested in ways to engender trust in this population, including investments in medicolegal partnerships, avoiding asking about immigration status, and improving telehealth and home visiting capacities.^35^

## Conclusions

In this study, there was a significant decrease noted in the number of completed ambulatory visits among a group of predominantly undocumented patients compared with Medicaid patients following an increase in anti-immigrant rhetoric associated with the 2016 presidential campaign. This and reported decreases in health services use among immigrants in other regions of the US add to the hypothesis that changes in such rhetoric might be an important risk factor for attrition from primary care.

## Notes

Supplement. **eAppendix.** Note on Quarterly Adjusted Averages **eTable 1.** Breakdown of Cohort by Age Groups **eTable 2.** Difference-in-Differences for Completed Primary Care Visits (Restricted to Those With Any Scheduled Visits in 2014) **eTable 3.** Difference-in-Differences for Completed Primary Care Visits for Adults and Children Who Were Not Hospitalized During the 2014-2018 Period **eTable 4.** Difference-in-Differences for Completed Primary Care Visits (Restricted to Those Between the Ages of 19 and 69 as of 2018) **eTable 5.** Difference-in-Differences for Completed Primary Care Visits (Estimated Under Gaussian Distribution) **eTable 6.** Difference-in-Differences of Completed Primary Care Visits for Latino Children Medicaid Recipients Compared to Non-Latino Medicaid Children Controls for Completed Primary Care Visits (Restricted to Those Between the Ages of 5 and 18 as of 2018) **eTable 7.** Secondary Outcome Difference-in-Differences **eTable 8.** Difference-in-Differences for Emergency Room Admission Rates **eTable 9.** Difference-in-Differences Among Primary and Secondary Outcomes for All Children Under Age 18 as of 2018 **eTable 10.** Unadjusted Estimates and Standard Deviations of Primary and Secondary Outcomes **eFigure 1.** Study Selection **eFigure 2.** Adjusted Trends in Scheduled Primary Care Appointments Among Adults **eFigure 3.** Adjusted Trends in Scheduled Primary Care Appointments Among Children e**Figure 4.** No Show/Cancellation Rate Among Adults e**Figure 5.** Adjusted Trends in No Show/Cancellation Rate Among Children **eFigure 6.** Number of Community Arrests by Immigration and Customs Enforcement in Baltimore City via the Syracuse University Transactional Records Access Clearinghouse
