# Errors in Key Points and Results

**DOI:** 10.1001/jamanetworkopen.2021.7292

**Published:** 2021-03-26

In the Original Investigation titled “Changes in Health Care Use Among Undocumented Patients, 2014-2018,”^1^ published March 5, 2021, there were errors in the percentage values in Key Points and wording errors and a numeric error in the second paragraph of the Results section. In the Key Points, percentage values given in sequence as 34.5% and 43.3% should have been reversed. In the first paragraph of the Results section, 2 parenthetical phrases starting “adjust postperiod mean” should have been “adjusted postperiod mean.” The mean value given as “1553”should have been “155.3.” This article has been corrected.^1^
