# Supplementary material for: Changes in Health Care Use Among Undocumented Patients, 2014-2018
Source: JAMA Netw Open. 2021 Mar 5;4(3):e210763. doi: 10.1001/jamanetworkopen.2021.0763 (PMC7936260; doi:10.1001/jamanetworkopen.2021.0763)
Supplement: Supplement. — eAppendix. Note on Quarterly Adjusted Averages eTable 1. Breakdown of Cohort by Age Groups eTable 2. Difference-in-Differences for Completed Primary Care Visits (Restricted to Those With Any Scheduled Visits in 2014) eTable 3. Difference-in-Differences for Completed Primary Care Visits for Adults and Children Who Were Not Hospitalized During the 2014-2018 Period eTable 4. Difference-in-Differences for Completed Primary Care Visits (Restricted to Those Between the Ages of 19 and 69 as of 2018) eTable 5. Difference-in-Differences for Completed Primary Care Visits (Estimated Under Gaussian Distribution) eTable 6. Difference-in-Differences of Completed Primary Care Visits for Latino Children Medicaid Recipients Compared to Non-Latino Medicaid Children Controls for Completed Primary Care Visits (Restricted to Those Between the Ages of 5 and 18 as of 2018) eTable 7. Secondary Outcome Difference-in-Differences eTable 8. Difference-in-Differences for Emergency Room Admission Rates eTable 9. Difference-in-Differences Among Primary and Secondary Outcomes for All Children Under Age 18 as of 2018 eTable 10. Unadjusted Estimates and Standard Deviations of Primary and Secondary Outcomes eFigure 1. Study Selection eFigure 2. Adjusted Trends in Scheduled Primary Care Appointments Among Adults eFigure 3. Adjusted Trends in Scheduled Primary Care Appointments Among Children eFigure 4. No Show/Cancellation Rate Among Adults eFigure 5. Adjusted Trends in No Show/Cancellation Rate Among Children eFigure 6. Number of Community Arrests by Immigration and Customs Enforcement in Baltimore City via the Syracuse University Transactional Records Access Clearinghouse [file jamanetwopen-e210763-s001.pdf]

Supplementary Online Content

Nwadiuko J, German J, Chapla K, et al. Changes in health care use among undocumented patients, 2014-2018. *JAMA Netw Open*. 2021;4(3):e210763. doi:10.1001/jamanetworkopen.2021.0763

eAppendix. Note on Quarterly Adjusted Averages

eTable 1. Breakdown of Cohort by Age Groups

eTable 2. Difference-in-Differences for Completed Primary Care Visits (Restricted to Those With Any Scheduled Visits in 2014)

eTable 3. Difference-in-Differences for Completed Primary Care Visits for Adults and Children Who Were Not Hospitalized During the 2014-2018 Period

eTable 4. Difference-in-Differences for Completed Primary Care Visits (Restricted to Those Between the Ages of 19 and 69 as of 2018)

eTable 5. Difference-in-Differences for Completed Primary Care Visits (Estimated Under Gaussian Distribution)

eTable 6. Difference-in-Differences of Completed Primary Care Visits for Latino Children Medicaid Recipients Compared to Non-Latino Medicaid Children Controls for Completed Primary Care Visits (Restricted to Those Between the Ages of 5 and 18 as of 2018)

eTable 7. Secondary Outcome Difference-in-Differences

eTable 8. Difference-in-Differences for Emergency Room Admission Rates

eTable 9. Difference-in-differences Among Primary and Secondary Outcomes for All Children Under Age 18 as of 2018

eTable 10. Unadjusted Estimates and Standard Deviations of Primary and Secondary Outcomes

eFigure 1. Study Selection

eFigure 2. Adjusted Trends in Scheduled Primary Care Appointments Among Adults

eFigure 3. Adjusted Trends in Scheduled Primary Care Appointments Among Children

e**Figure 4.** No Show/Cancellation Rate Among Adults

e**Figure 5.** Adjusted Trends in No Show/Cancellation Rate Among Children

eFigure 6. Number of Community Arrests by Immigration and Customs Enforcement in Baltimore City via the Syracuse University Transactional Records Access Clearinghouse

This supplementary material has been provided by the authors to give readers additional information about their work.

© 2021 Nwadiuko J et al. *JAMA Network Open.*

eAppendix. Note on Quarterly Adjusted Averages

Predicted estimates for Quarterly Adjusted Averages are as derived from the following model:

$$\ln(\mu_{ij}) = \beta_0 + \beta_1 Cohort_i + \beta_2 Quarter_{ij} + \beta_3 Cohort_i x Quarter_{ij} + \beta_4 Birthyear_i + \beta_5 Gender_i$$
$$+ \beta_6 Elixhauser_i + \beta_7 Gender_i \times Quarter_{ij} + \beta_8 BirthYear_i \times Quarter_{ij}$$
$$+ \ln(time_{ij})$$

Where *Cohort* represents either the Medicaid control group or Medicaid-ineligible group; *Age* represents Age as of 2018, *Gender* represents self-reported gender, and *Elixhauser* represents the Elixhauser Comorbidity weight. The offset for no-show/cancellation rates is *ln(number of scheduled visits)* instead of *ln*(*time).* The outcome μ specifies the number of encounters (or number of missed visits in the case of no-show/cancellations), the *i* subscript refers to each individual, and the j subscript specifies the quarter.

© 2021 Nwadiuko J et al. *JAMA Network Open.*

eTable 1: Breakdown of cohort by age groups

Ages (as of 2018)	Control	%	Cohort	%	Total
**0-5[a]**	2,906	13.4%	5	0.3%	**2,911**
**6-9**	3,971	18.4%	29	1.9%	**4,000**
**10-19**	7287	33.7%	161	10.7%	**7448**
**20-29**	3182	14.7%	231	15.3%	**3413**
**30-39**	1242	5.8%	404	26.8%	**1646**
**40-49**	922	4.3%	352	23.4%	**1274**
**50-59**	1,128	5.2%	186	12.3%	**1314**
**60-69**	857	3.9%	86	5.8%	**943**
**70-99**	121	0.6%	52	3.4%	**173**
**Total**	**21625**	**100%**	**1,508**	**100%**	**23,122**

[a] Excluded from all but supplementary analyses. See Methods (Selection Criteria) for details.

eTable2. Difference-in-differences for Completed Primary Care Visits (restricted to those with any scheduled visits in 2014)

	Average Adjusted Annual Number of Encounters (per 100 people)					
	Medicaid-Ineligible Patients		Medicaid-Patients		Difference in Differences Estimate (Incident Rate Ratio)	
	Pre	Post	Pre	Post	Estimate (95% CI)	p
Adults	215.6	115.2	208.3	157.5	0.7 (0.7-0.8)	<0.001
Children	237.8	107.1	180.0	127.4	0.6 (0.5-0.8)	<0.001

© 2021 Nwadiuko J et al. *JAMA Network Open.*

eTable 3: Difference-in-differences for Completed Primary Care Visits for Adults and Children Who Were Not Hospitalized During the 2014-2018 Period

	Average Adjusted Annual Number of Encounters (per 100 people)					
	Medicaid-Ineligible Patients		Medicaid Patients		Difference in Differences Estimate (Incident Rate Ratio)	
	Pre	Post	Pre	Post	Estimate (95% CI)	p
Adults	171.3	104.8	175.2	132.8	0.8 (0.7-0.9)	<0.001
Children	185.4	108.8	168.3	122.1	0.8 (0.7-1.0)	0.02

© 2021 Nwadiuko J et al. *JAMA Network Open.*

eTable 4: Difference-in-differences for Completed Primary Care Visits (restricted to those between the ages of 19 and 69 as of 2018)

Average Adjusted Annual Number of Encounters (per 100 people)					
Medicaid-Ineligible Patients (per 100 persons)		Medicaid Patients (per 100 persons)		Difference in Differences Estimate (Incident Rate Ratio)	
Pre	Post	Pre	Post	Estimate (95% CI)	P
178.5	117.5	192.6	154.1	0.8 (0.8-0.9)	<0.001

© 2021 Nwadiuko J et al. *JAMA Network Open.*

eTable 5: Difference-in-differences for Completed Primary Care Visits (estimated under Gaussian distribution)

	Average Adjusted Annual Number of Encounters (per 100 people)					
	Medicaid-Ineligible Patients		Medicaid-Patients		Difference in Differences Estimate	
	Pre	Post	Pre	Post	Estimate (95% CI)	p
Adults	184.2	132.4	208.2	173.4	-0.2 (-0.2 - -0.1)	<0.001
Children	192.7	114.9	178.4	128.4	-0.3 (-0.5 - -0.1)	<0.001

© 2021 Nwadiuko J et al. *JAMA Network Open.*

eTable 6: Difference-in-differences of Completed Primary Care visits for Latino children Medicaid recipients compared to non-Latino Medicaid children controls for Completed Primary Care Visits (restricted to those between the ages of 5 and 18 as of 2018)

Average Adjusted Annual Number of Encounters (per 100 people)					
Latino Medicaid recipients		Medicaid Patients		Difference in Differences Estimate (Incident Rate Ratio)	
Pre	Post	Pre	Post	Estimate (95% CI)	P
196.2	141.2	167.5	122.3	1.0 (1.0- 1.0)	0.47

© 2021 Nwadiuko J et al. *JAMA Network Open.*

eTable 7 Secondary Outcome Difference-in-Differences

	Average Adjusted Annual Number of Encounters (per 100 people)					
	Medicaid-Ineligible Patients[a]		Medicaid Patients[a]		Difference in Differences Estimate (Incident Rate Ratio)	
	Pre	Post	Pre	Post	Estimate (95% CI)	p
**Scheduled Primary Care Visits**						
Adults	297.2	230.4	402.8	364.2	0.8 (0.7-0.9)	<0.001
Children	263.2	167.1	321	235	0.8 (0.7-1.0)	0.014
**No Show/Cancellation Rates**						
Adults	39.3	40.9	51.4	53.3	1.0 (0.9-1.1)	0.97
Children	28.8	31.4	46.4	46.3	1.1 (0.9-1.3)	0.31

[a]Scheduled visit values are per 100 persons. No-show/cancellation rates are represented as percentages of all scheduled visits.

© 2021 Nwadiuko J et al. *JAMA Network Open.*

eTable 8. Difference-in-differences for Emergency Room Admission Rates

	Average Adjusted Annual Number of Encounters (per 100 people)					
	Medicaid-Ineligible Patients (%)		Medicaid Patients (%)		Difference in Differences Estimate (Incident Rate Ratio)	
	Pre	Post	Pre	Post	Estimate (95% CI)	p
Adults	3.8	4.7	7.9	6.6	1.5 (1.0-2.3)	0.064
Children	1.6	4.6	2.9	3.8	2.2 (0.5-9.0)	0.29

© 2021 Nwadiuko J et al. *JAMA Network Open.*

eTable 9. Difference-in-differences among Primary and Secondary Outcomes for all children under age 18 as of 2018

	Average Adjusted Annual Number of Encounters (per 100 people)					
	Medicaid-Ineligible Patients[a]		Medicaid Patients[a]		Difference in Differences Estimate (Incident Rate Ratio)	
	Pre	Post	Pre	Post	Estimate (95% CI)	p
Completed Visits	285.8	127.2	256.8	147.9	0.8 (0.65-0.92)	0.003
Scheduled Visits	335.8	172.4	409.8	259.5	0.8 (0.68-0.96)	0.016
No Show/Cancellation Rates	27.4	31.5	44.1	45.4	1.1 (0.95-1.32)	0.182
Emergency Room Visits	6.7	14.4	42.4	36.6	2.3 (1.10-4.95)	0.027
Inpatient Discharges	5.7	3.3	3.6	2.4	0.9 (0.15- 5.19)	0.90

[a]Scheduled visit values are per 100 persons. No-show/cancellation rates are represented as percentages of all scheduled visits.

eTable 10. Unadjusted estimates and standard deviations of Primary and Secondary Outcomes

	Average Annual Number of Encounters (per 100 people)				
	Medicaid-Ineligible Patients[a]		Medicaid Patients[a]		Unadjusted Incident Rate Ratio
	Pre (st. dev.)	Post (st. dev.)	Pre (st. dev.)	Post (st. dev.)	
Completed Visits--Adults	236.6 (216.3)	334.2 (448.0)	308.4 (317.8)	511.6 (668.5)	0.9
Completed Visits—Children	237.0 (162.3)	290.3 (293.2)	260.3 (206.8)	380 (326.3)	0.8
Emergency Room Visits—Adults	18.7 (66.6)	37.2 (113.3)	91.3 (304.2)	166.8 (502.3)	1.1
Emergency Room Visits-Children	7.3 (34.2)	35.7 (131.1)	47.8 (121.2)	86.8 (184.8)	2.7
Inpatient Discharges-Adults	12.1 (68.2)	8.7 (39.6)	29.2 (119.7)	40.6 (157.1)	0.5
Inpatient Discharges—Children	5.4 (37.0)	12.1 (105.2)	5.4 (41.3)	8.2 (53.0)	1.5

© 2021 Nwadiuko J et al. *JAMA Network Open.*

eFigure 1. Study Selection

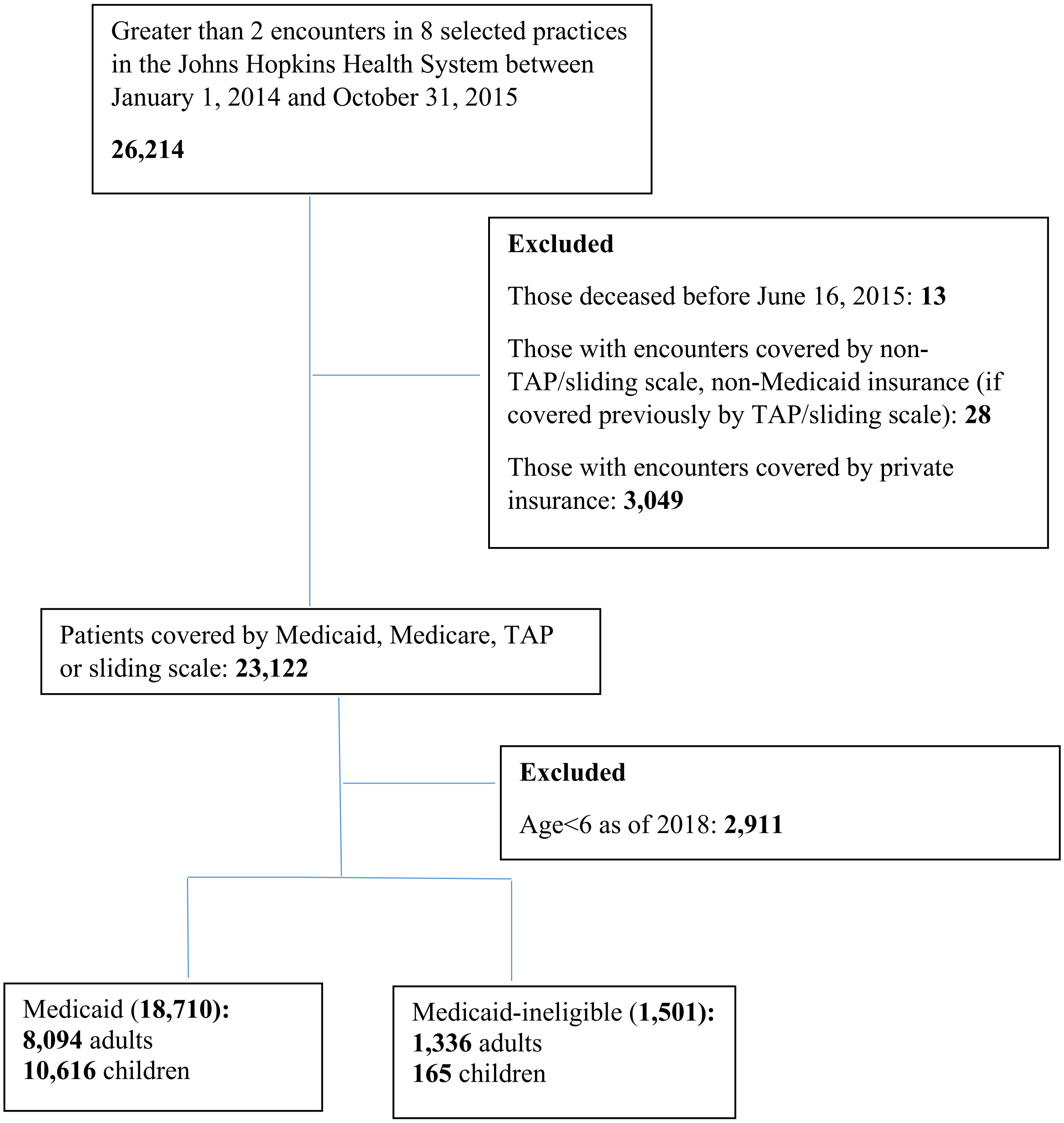

© 2021 Nwadiuko J et al. *JAMA Network Open.*

eFigure 2 Adjusted Trends in Scheduled Primary Care Appointments among Adults

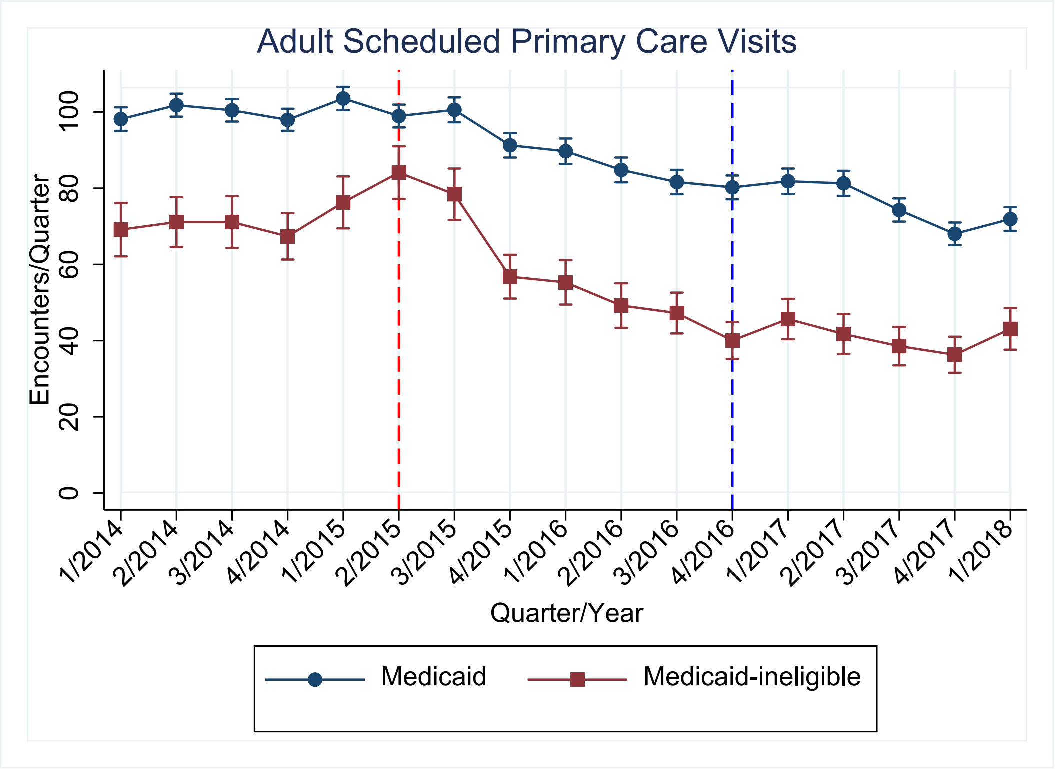

© 2021 Nwadiuko J et al. *JAMA Network Open.*

eFigure 3 Adjusted Trends in Scheduled Primary Care Appointments among Children

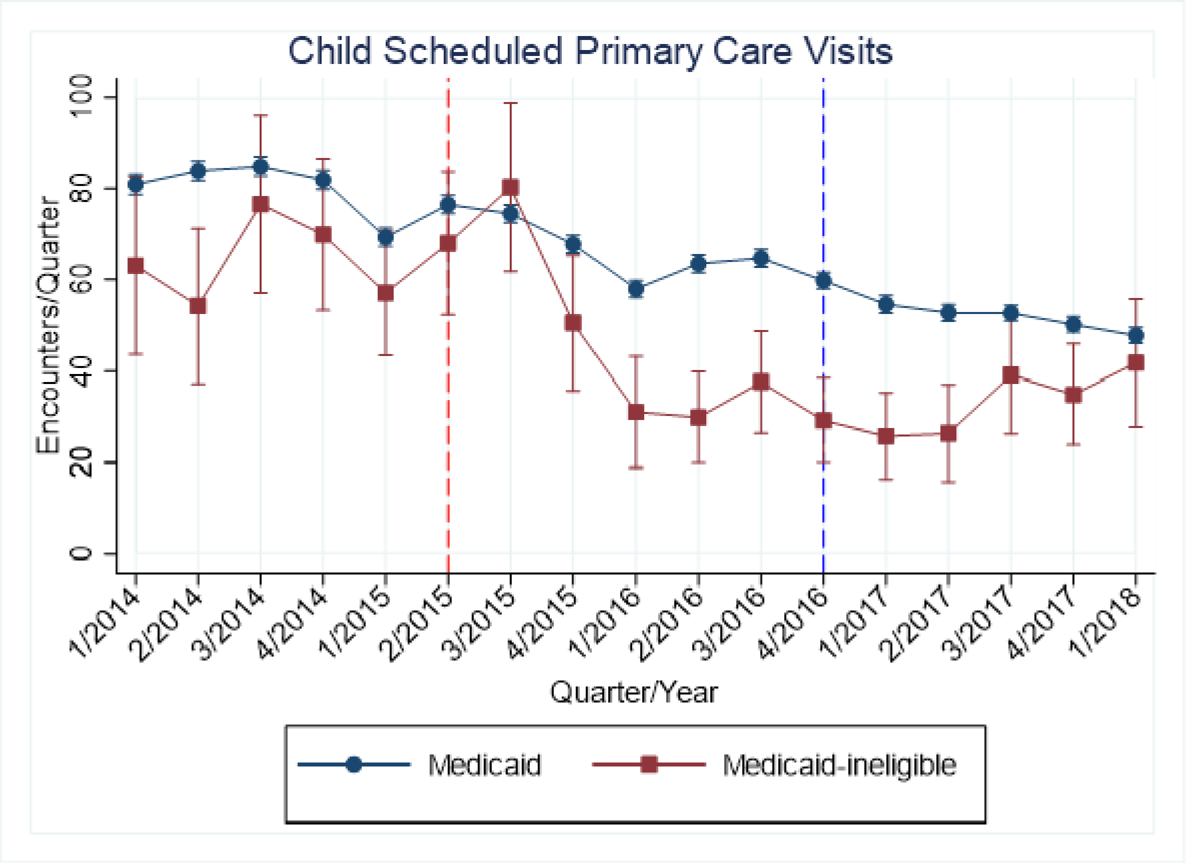

--Announcement of Donald Trump's candidacy for president

--2016 General Presidential Election

© 2021 Nwadiuko J et al. *JAMA Network Open.*

eFigure 4 No Show/Cancellation Rate Among Adults

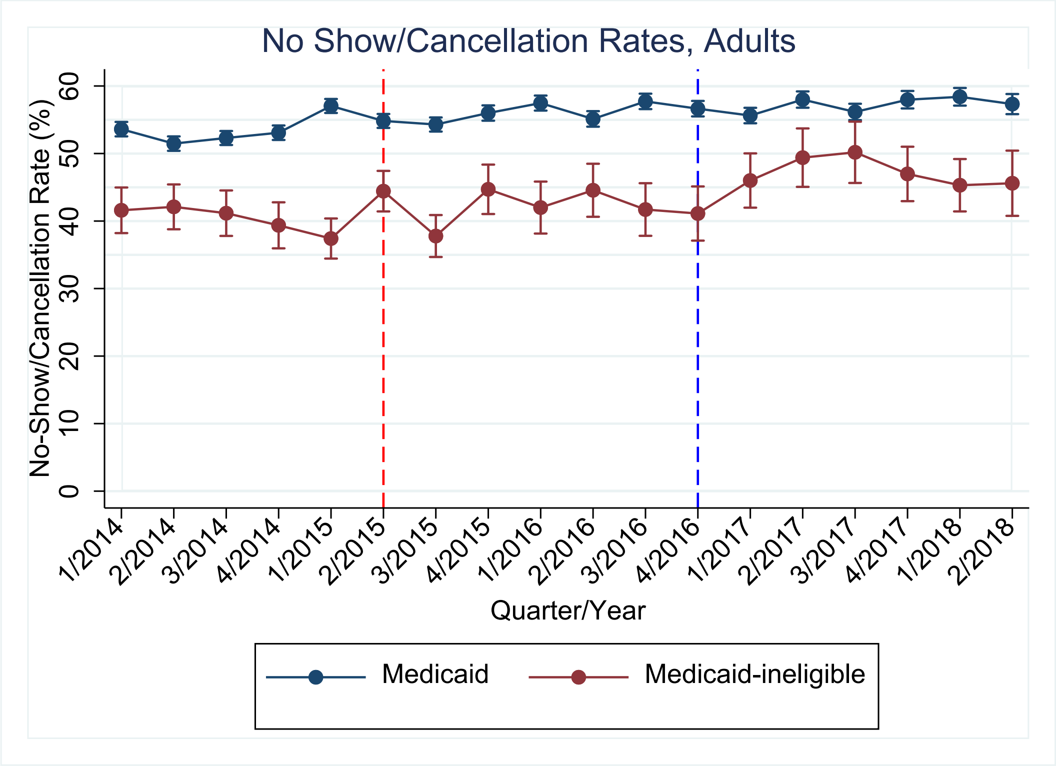

--Announcement of Donald Trump's candidacy for president

--2016 General Presidential Election

© 2021 Nwadiuko J et al. *JAMA Network Open.*

eFigure 5 Adjusted Trends in No-Show Cancellation Rate among Children

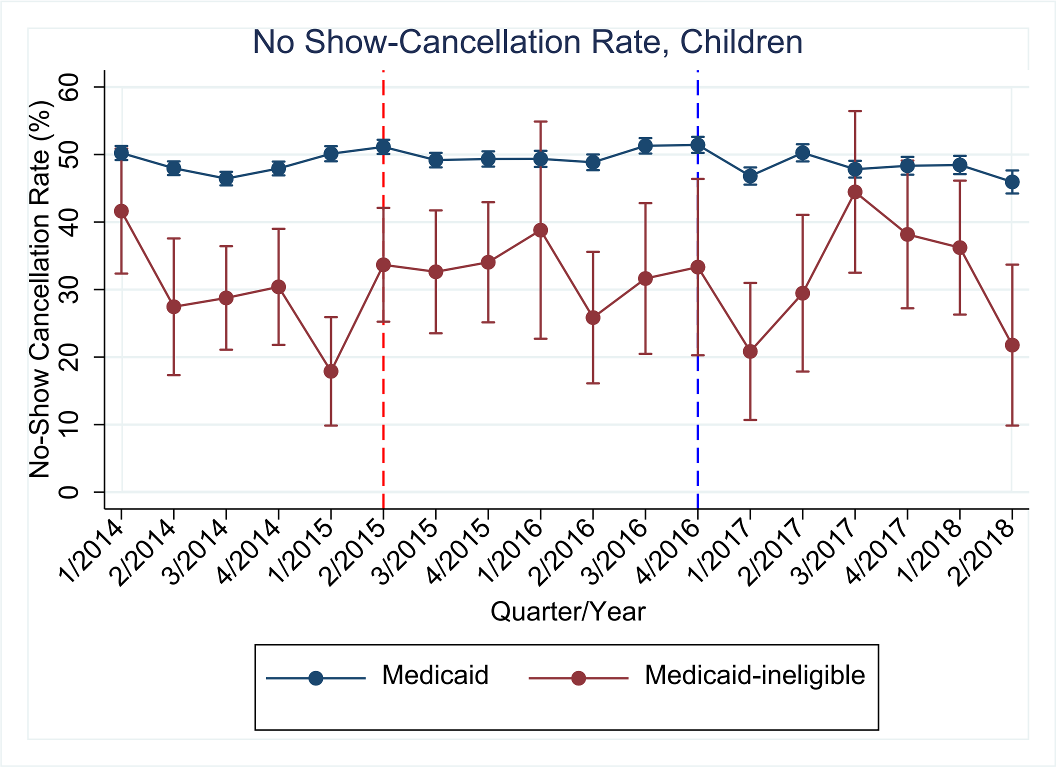

--Announcement of Donald Trump's candidacy for president

--2016 General Presidential Election

© 2021 Nwadiuko J et al. *JAMA Network Open.*

eFigure 6: Number of community arrests by Immigration and Customs Enforcement in Baltimore City via the Syracuse University Transactional Records Access Clearinghouse

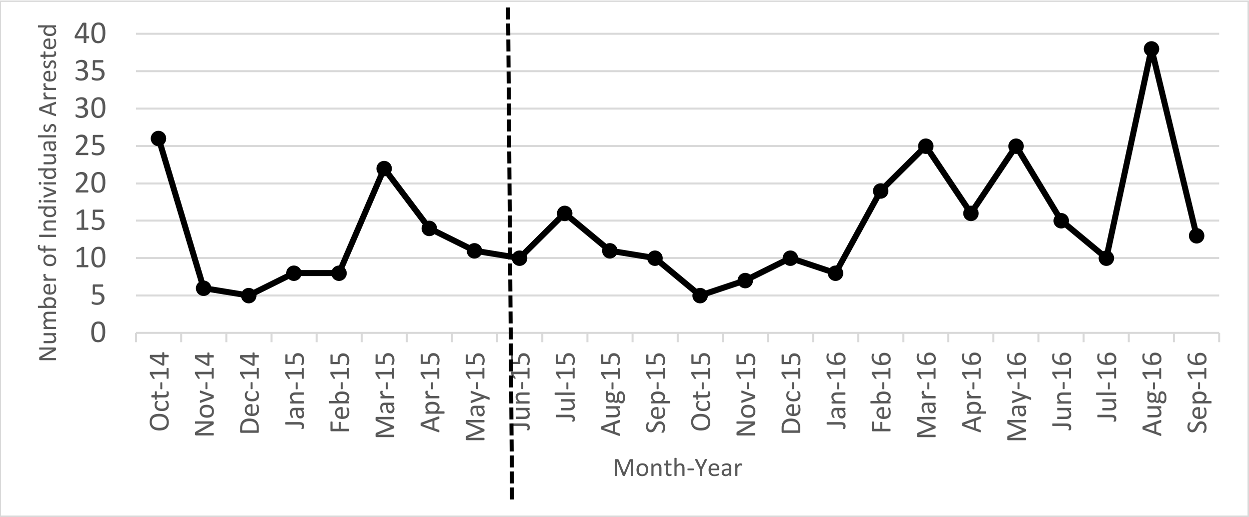

© 2021 Nwadiuko J et al. *JAMA Network Open.*
